# Impact of a Community Health Worker (CHW) Home Visiting Intervention on Any and Adequate Prenatal Care Among Ethno-Racially Diverse Pregnant Women of the US Southwest

**DOI:** 10.1007/s10995-022-03506-2

**Published:** 2022-10-21

**Authors:** Kelly McCue, Samantha Sabo, Patrick Wightman, Matthew Butler, Vern Pilling, Dulce Jiménez, Rebecca Annorbah, Sara Rumann

**Affiliations:** 1 Northern Arizona University Center for Health Equity Research, PO Box 4065, 86001 Flagstaff, AZ USA; 2 University of Arizona Center for Population Science and Discovery, P.O. Box 245024, 85724 Tucson, AZ USA; 3 Department of Economics, Brigham Young University, 435 Crabtree Technology Building, 84602 Provo, UT USA; 4 University of Arizona Center for Biomedical Informatics and Biostatistics, PO Box 210242, 85721 Tucson, AZ USA; 5 Arizona Department of Health Services, Bureau of Women’s and Children’s Health, 150 North 18th Avenue, Suite 320, 85007 Phoenix, AZ USA

**Keywords:** Home visiting, community health worker, prenatal care, propensity score matching

## Abstract

**Objectives:**

Social and structural barriers drive disparities in prenatal care utilization among minoritized women in the United States. This study examined the impact of Arizona’s Health Start Program, a community health worker (CHW) home visiting intervention, on prenatal care utilization among an ethno-racially and geographically diverse cohort of women.

**Methods:**

We used Health Start administrative and state birth certificate data to identify women enrolled in the program during 2006–2016 (n = 7,117). Propensity score matching was used to generate a statistically-similar comparison group (n = 53,213) of women who did not participate in the program. Odds ratios were used to compare rates of prenatal care utilization. The process was repeated for select subgroups, with post-match regression adjustments applied where necessary.

**Results:**

Health Start participants were more likely to report any (OR 1.24, 95%CI 1.02–1.50) and adequate (OR 1.08, 95%CI 1.01–1.16) prenatal care, compared to controls. Additional specific subgroups were significantly more likely to receive any prenatal care: American Indian women (OR 2.22, 95%CI 1.07–4.60), primipara women (OR 1.64, 95%CI 1.13–2.38), teens (OR 1.58, 95%CI 1.02–2.45), women in rural border counties (OR 1.45, 95%CI 1.05–1.98); and adequate prenatal care: teens (OR 1.31, 95%CI 1.11–1.55), women in rural border counties (OR 1.18, 95%CI 1.05–1.33), primipara women (OR 1.18, 95%CI 1.05–1.32), women with less than high school education (OR 1.13, 95%CI 1.00-1.27).

**Conclusions for Practice:**

A CHW-led perinatal home visiting intervention operated through a state health department can improve prenatal care utilization among demographically and socioeconomically disadvantaged women and reduce maternal and child health inequity.

**Supplementary Information:**

The online version contains supplementary material available at 10.1007/s10995-022-03506-2.

## Significance

*What is already known on this subject?* Prenatal care is associated with improved maternal and child health outcomes, but significant social and structural determinants affect utilization of services among minority women in the US. Home visiting programs have contributed to early initiation of and recommended frequency of prenatal care among health disparate populations.

*What does this study add?* Most empirical studies of prenatal home visitation programs include teams with licensed health professionals. Arizona’s Health Start Program is one of few home visiting interventions associated with increased prenatal care utilization in which CHWs are the primary interventionists.

Impact of a community health worker (CHW) home visiting intervention on any and adequate prenatal care among ethno-racially diverse pregnant women of the US Southwest.

**Supplementary Information:**

The online version contains supplementary material available at 10.1007/s10995-022-03506-2.

## Introduction

In this paper, we describe the relationship between participation in the Health Start Program and the utilization of prenatal care (PNC), including among participant subgroups associated with higher risks of adverse health outcomes, based on demographic or socioeconomic characteristics. This study is part of a larger research agenda evaluating the effect of the Arizona Health Start Program on newborn health, maternal healthcare utilization, and early child health from 2006 to 2016 (Sabo et al., 2019, 2021).

Arizona is one of the largest US states by geographical size but ranks fourteenth in population due to its rural geography. Compared to the US, Arizona has higher proportions of Latino (30.9% vs. 17.8%) and American Indian (5% vs. 1%) residents, but fewer African Americans (5% vs. 13%) (ADHS, 2016). The demography is in part attributable to an international border shared with Mexico and 21 federally recognized American Indian Nations (ADHS, 2015). Nearly 25% of the population lives in rural areas, where the poverty rate is almost double the national rate (ADHS, 2015). 20% of Arizonans live along the 370-mile Arizona-Mexico border region, where PNC outcomes are worse compared to the interior regions (McDonald et al., 2015). The teen birth rate in Arizona exceeded the US average (29.5 vs. 20.3 per 1,000) in 2016, with rates highest among American Indian and Latina teens (ADHS, 2016).

Maternal and child health inequities disparately affect Latina and American Indian women in Arizona and the US compared to non-Hispanic white women (Office of Minority Health, n.d.). Social, cultural, and structural barriers to PNC are important factors. Barriers to PNC among Latina and American Indian women include young maternal age and primiparity, socioeconomic stressors, difficulty paying for care (e.g. limited health insurance), limited access to healthcare (e.g. transportation, geography), demanding work schedules, lack of social support, language barriers, and under-resourced or rural health systems (Johnson, 2020; Selchau et al., 2017; Shaffer, 2002). Living in medically underserved areas, perceived discrimination by providers, historical trauma (including devastating forced sterilization and infant separation policies), and limited access to culturally sensitive providers all contribute to distrust of the healthcare system and decreased use of PNC services among Latina and American Indian women in the US (Johnson, 2020; NPWF 2019; Raglan et al., 2016; Selchau et al., 2017; Shaffer, 2002).

Appropriate PNC utilization is linked to improved pregnancy and birth outcomes (Vintzileos et al., 2002) and, as an early intervention, can subsequently reduce the cost burden to families and health systems (Clements et al., 2007) and positively impact the life-course health of women and their families over generations.

## Objective

We assess whether participation in the Health Start Program between 2006 and 2016 was associated with better PNC outcomes among an ethno-racially and geographically diverse cohort of women in Arizona. We used methodologies that meet rating criteria for “moderate” effectiveness (the highest rating for which non-experimental comparison group designs are eligible), established by Home Visiting Evidence of Effectiveness (HomVEE) guidelines (HomVEE, n.d.).

## Methods

### The Arizona Health Start Program

Health Start was created in 1984 in response to the rising rates of inadequate PNC among pregnant Latina im/migrant farm workers in the border region of southern Arizona. In 1994, under state legislation and the administration of the Arizona Department of Health Services (ADHS) Bureau of Women’s and Children’s Health, the program was expanded to all 14 counties to serve a broader population of women at higher risk for adverse birth outcomes. The program aims to improve maternal and child health through several goals, including increasing PNC use and reducing the incidence of low birthweight infants. Recent evaluations of Health Start (Sabo et al., 2019, 2021; Hussaini et al., 2011), find significant reductions in the likelihood of low birthweight outcomes and preterm birth among women who participated in the program.

Women are eligible for Health Start if they are pregnant or have a child under age two. As a community-based outreach program, Health Start employs community health workers (CHWs) to identify, screen, and enroll pregnant women early in their pregnancies and provide perinatal education, referrals, and advocacy services promoting maternal and child health (MCH). Health Start CHWs reflect the ethnic, cultural, and socioeconomic characteristics of the communities they serve and act as the primary interventionists (Sabo et al., 2019, 2021). They receive comprehensive training in CHW Core Competencies, including cultural mediation and provision of culturally appropriate health education and information, care coordination and system navigation, social support, advocacy and capacity building, and outreach (Rosenthal et al., 2016). CHWs undergo several hours of training to develop program-specific skills and education on home visiting, community outreach, emotional support, and various perinatal and infant health topics, detailed in Sabo et al., (2019).

### Summary of Prenatal Education Within Health Start

Health Start CHWs assist with accessing appropriate PNC services and provide prenatal education, referrals, and advocacy for clients. Prenatal education includes information about the importance of early and continuous PNC from a medical provider, what to expect in pregnancy and labor, healthy behaviors during pregnancy (e.g. nutrition, physical activity), warning signs in pregnancy, and other related topics based on client need. CHWs screen for substance use (i.e. alcohol, tobacco, drug), and signs of perinatal depression, partner abuse, and other social determinants of MCH-related behaviors as needed. CHWs follow their clients through their pregnancy, with up to four (4) prenatal home visits per month, and may attend labor and delivery. While most CHW services are delivered in prescheduled home visits, these may also be provided at community locations or alternative living situations such as rehabilitation centers, jails, inpatient treatment centers, homeless shelters, and alternative high schools for pregnant teens. Health Start CHWs build rapport within their communities and with their clients by leveraging their trusted relationships and knowledge of resources to meet the needs of high-risk pregnant women and their families (Sabo et al., 2017).

### Outcome Measures

Our outcome measures are derived from the Adequacy of Prenatal Care Utilization (APNCU) index, summarized in Table 1 (Kotelchuck, 1994). The APNCU Index assesses (a) adequacy of initiation of PNC (month PNC began), and (b) adequacy of received services, measured as the ratio of actual (reported) number of prenatal visits to the recommended number of visits per American College of Obstetricians and Gynecologist (ACOG) guidelines: monthly visits through week 28, biweekly visits through week 36, and weekly visits thereafter, adjusted for date of initiation of PNC (American Academy of Pediatrics and ACOG, 2017). This index generates four levels: Inadequate, Intermediate, Adequate, and Adequate Plus. The primary measures used for the present analysis are the utilization of any PNC (vs. no PNC) and the receipt of at least Adequate PNC (Adequate or Adequate Plus vs. Intermediate and Inadequate PNC). For completeness we also look at each endpoint of the index: Adequate Plus PNC (vs. Adequate, Intermediate, and Inadequate) and Inadequate PNC (vs. Intermediate, Adequate and Adequate Plus).

**Table 1:** Summary of the Adequacy of Prenatal Care Utilization (APNCU) Index

Adequate Plus	Prenatal care begun by the 4th month (of pregnancy) and 110% or more of recommended (prenatal care) visits received
Adequate	Prenatal care begun by the 4th month and 80–109% of recommended visits received
Intermediate	Prenatal care begun by the 4th month and 50–79% of recommended visits received
Inadequate	Prenatal care begun after the 4th month or less than 50% of recommended visits received

Summary from: Kotelchuck 1994. An evaluation of the Kessner Adequacy of Prenatal Care Index and a proposed Adequacy of Prenatal Care Utilization Index. American Journal of Public Health 84, 1414_1420, 10.2105/AJPH.84.9.1414

### Study Population & Research Design

**Fig. 1:**
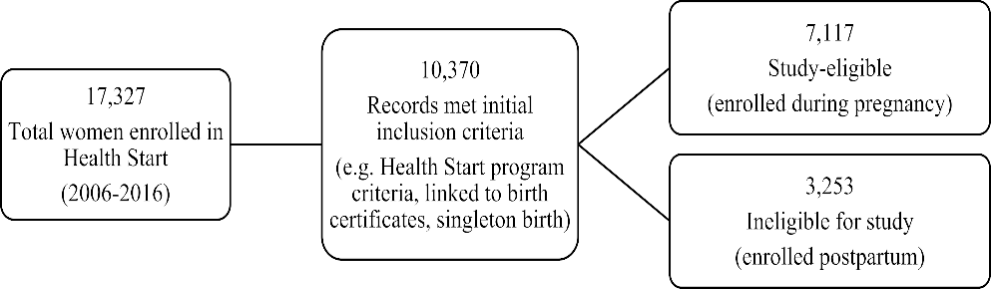
Intervention Group Selection

Our initial study population includes all births reported to the Arizona Vital Records Birth Database between 2006 and 2016 (the year the present study was initiated). The intervention group was identified by linking Health Start enrollment records to state birth certificate records. Details of the linking process are found in the Health Start evaluation protocol (Sabo et al., 2019). Intervention group eligibility was limited to participants who (1) were enrolled prior to giving birth and (2) linked to a certificate for live singleton birth. These criteria resulted in 7,117 women in the Health Start intervention group (Fig. 1). This research follows prevailing ethical principles and obtained a waiver of informed consent, approved by the University of Arizona and ADHS Human Subjects Review Boards. This is not based upon clinical study or patient data.

Given the criteria that must be met in order to participate in Health Start, the intervention group is (on average) demographically and socio-economically different from the population of Arizona women as a whole. Accordingly, we used propensity score matching (PSM) to identify a statistically appropriate synthetic control group from the birth certificate records. PSM restricts intervention-control comparisons to women with statistically similar baseline (i.e. pre-intervention) characteristics. Variables used to match potential controls to the intervention group include characteristics that determine Health Start eligibility, i.e. demographic, socioeconomic (SES), and medical risk factors. Other matching variables include additional demographic characteristics, and geographic and time-period indicators. These restrict candidate matches to women who gave birth within the same locations and calendar years and implicitly accounts for broad macroeconomic conditions (Dehejia & Wahba, 2002).

All variables used for the matching and outcome comparisons were derived from birth certificate records. While the information from these records is limited, this registry is one of few available large-scale resources from which common secondary data for both intervention and control groups may be obtained. Propensity scores were estimated via logistic regression and used to identify each Health Start participant’s nearest statistical neighbor(s). Multiple nearest-neighbor matches (i.e. ties) were permitted based on propensity score. In these cases, software-generated weights account for the number of control-group matches and the number of intervention group members with the same propensity score to mimic a one-to-one match. This resulted in 53,213 women in the synthetic control group (unweighted).

PSM is a non-experimental comparison group design. Upon meeting criteria prescribed in the What Works Clearinghouse (WWC) Standards Handbook (Version 4.1, 2020), these designs qualify for HomVEE’s “moderate” effectiveness rating (HomVEE, n.d.; What Works Clearinghouse 2020). These criteria concern the establishment of baseline equivalence, so that differences in outcomes can be clearly attributed to the intervention itself. In order to qualify, the standardized differences (SD) between variables used for matching the intervention and control groups must be below 0.25. For those groups where all SDs fall below 0.05, no further adjustment is necessary. Where the SD is above 0.05 and below 0.25, further (post-matching) adjustment is required.

We repeated the PSM matching process and subsequent outcome comparisons for six high-risk and/or disadvantaged subgroups: Latina and American Indian women, women residing in rural Arizona-Mexico border counties, women with less than a high school/GED education, teen (under age 20) mothers, and primipara women. While these groups are not mutually exclusive, these individual demographic and socioeconomic characteristics are important Health Start eligibility criterion, and markers of historical under-service by maternal and child health programs, historically low engagement in prenatal care, and/or increased risk for adverse birth outcomes (e.g. preterm birth, low birthweight). Table 2 reports the PNC outcomes for these groups. While rates of any PNC exceed 95% for all groups, rates of subsequent PNC among these groups are worse compared to the State averages (with the exception of primipara women). With respect to at-least-adequate care, the differences range from 6% points (Latina women) to nearly 17% points (American Indian women).

**Table 2:** Prenatal Care (PNC) Outcomes, Arizona State Rates (2006–2016)

	Statewide	Primipara	Rural Border	Latina	American Indian	Less than High School	Teen	
Any PNC	97.9%	98.4%	95.4%	97.0%	97.2%	95.8%	96.8%	
< Adequate PNC^a^	15.3%	13.9%	25.1%	20.0%	27.0%	25.7%	24.6%	
≥ Adequate PNC^b^	71.3%	72.9%	62.8%	65.3%	54.7%	58.4%	59.9%	
Adequate Plus PNC	27.3%	27.7%	23.1%	24.8%	18.0%	22.7%	21.6%	

Outcomes based on APNCU Index categories (see Table 1)

a. Includes ‘Inadequate’ and ‘Intermediate’ categories

b. Includes ‘Adequate’ and ‘Adequate Plus’ categories

## Results

### Population Characteristics & Matching

Descriptive statistics for the variables used to generate the matched-control cohorts, for the state and Health Start cohorts and six subgroups, are reported in Tables 3a-3c. Individual demographic and health control variables included age, race/ethnicity and country of birth, marital and cohabiting status, a first birth indicator, and presence of any reported pre-existing health conditions considered risk factors (non-gestational diabetes, hypertension, and/or previous preterm birth). Direct SES measures included maternal education and the primary insurance payer. We also included individual controls for county of residence and calendar year. Given some characteristics are distributed differently within subgroups, models for these populations included additional interactions between covariates to achieve balance (not reported here, all results) available on request.

**Table 3a:** Matching Results (Baseline Equivalence) for Health Start Participants: Full Cohort & Primipara Subgroup

	Arizona Non-HSP	Full Cohort				Primipara				
		HSP	Matches	p-value	SD	HSP	Matches	p-value	SD	
N (Unweighted)	961,926	7,177	53,213			2,994	18,213			
**Maternal age**										
Age < 20	9.9	17.6	16.6	0.132	0.027	34.0	33.1	0.477	0.040	
Age 20–24	25.3	34.4	35.2	0.353		40.9	42.7	0.173		
Age 25–30 (ref)	34.0	28.4	28.4	0.985		18.0	17.8	0.840		
Age > 30	30.8	19.6	19.8	0.737		7.0	6.3	0.301		
**Race/ethnicity**										
White	42.4	24.0	23.8	0.754	0.022	27.2	27.5	0.772	0.030	
American Indian	6.0	11.8	11.8	0.938		9.8	10.1	0.666		
Latina	41.9	59.1	59.7	0.444		57.7	57.7	0.958		
Other race/ethnicity (ref)	9.7	5.1	4.7	0.216		5.3	4.7	0.286		
**Maternal nativity**										
US (ref)	73.6	68.6	68.4	0.774	0.007	77.6	79.0	0.210	0.034	
Mexico	18.8	27.9	28.2	0.696		19.3	18.3	0.337		
Outside US	7.6	3.5	3.4	0.819		3.1	2.7	0.399		
**Maternal education**										
Less than high school (ref)	21.7	32.8	33.1	0.624	0.028	27.7	26.9	0.528	0.035	
High school/GED	28.9	35.7	35.2	0.565		37.9	37.4	0.729		
Some post-secondary	25.1	23.8	23.8	0.969		25.8	26.2	0.724		
4-year degree or more	22.5	7.3	7.1	0.561		8.4	9.0	0.435		
Education missing	1.6	0.3	0.5	0.158		0.3	0.4	0.332		
**Insurance/payer**										
Private/commercial insurance	41.1	13.8	13.8	0.923	0.013	16.1	16.9	0.403	0.022	
Medicaid	53.8	82.5	82.7	0.758		80.4	79.7	0.497		
Other insurance (ref)	5.1	3.7	3.5	0.420		3.5	3.4	0.832		
Married	54.5	37.8	38.0	0.796	0.004	28.0	27.7	0.795	0.007	
Cohabiting	75.6	62.4	62.6	0.836	0.003	54.9	54.8	0.979	0.001	
Primipara	36.9	41.7	40.7	0.222	0.020	100.0	100.0	1.000		
Pre-existing health risk^1^	8.2	11.3	10.5	0.108	0.027	8.4	7.6	0.294	0.027	

HSP: Health Start Program; SD: Standardized Difference

1. Pre-existing health risks defined as presence of pre-existing (non-gestational) diabetes and hypertension, and/or previous preterm birth

All models control for median income at zip code level, county of residence, and birth year. Subgroup matching models may include additional interactions between controls to achieve baseline equivalence

**Table 3b:** Matching Results (Baseline Equivalence) for Health Start Participants: Rural Border Counties, Latina & American Indian Subgroups

	Rural Border Counties				Latina				American Indian			
	HSP	Matches	p-value	SD	HSP	Matches	p-value	SD	HSP	Matches	p-value	SD
N (Unweighted)	2,388	7,166			4,241	32,116			846	2,245		
**Maternal age**												
Age < 20	21.4	21.0	0.724	0.019	18.5	17.8	0.367	0.021	18.7	16.9	0.341	0.064
Age 20–24	37.6	38.3	0.592		33.4	34.0	0.566		35.7	37.9	0.339	
Age 25–30 (ref)	24.7	24.9	0.920		27.2	27.4	0.807		28.7	27.4	0.552	
Age > 30	16.2	15.8	0.664		20.9	20.9	0.936		16.9	17.7	0.653	
**Race/ethnicity**												
White	14.7	15.8	0.295	0.067	0.0	0.0		0.000	0.0	0.0		0.000
American Indian	0.6	0.3	0.087		0.0	0.0			100.0	100.0	1.000	
Latina	80.9	80.8	0.941		100.0	100.0	1.000		0.0	0.0		
Other race/ethnicity (ref)	3.8	3.1	0.206		0.0	0.0			0.0	0.0		
**Maternal nativity**												
US (ref)	68.3	70.0	0.217	0.041	51.8	52.1	0.744	0.018	99.9	99.2	0.046	0.109
Mexico	30.2	28.4	0.171		46.9	46.7	0.879		0.0	0.6	0.019	
Outside US	1.5	1.7	0.678		1.3	1.1	0.430		0.1	0.1	1.000	
**Maternal education**												
Less than high school (ref)	29.1	30.2	0.423	0.051	38.9	40.1	0.236	0.030	31.0	27.7	0.135	0.090
High school/GED	38.1	38.1	0.976		35.1	34.5	0.553		40.2	42.4	0.348	
Some post-secondary	25.4	24.8	0.617		19.5	19.2	0.742		26.4	27.9	0.477	
4-year degree or more	7.1	6.5	0.455		6.2	5.7	0.382		2.1	1.5	0.365	
Education missing	0.1	0.2	0.201		0.3	0.3	0.980		0.4	0.5	0.714	
**Insurance/payer**												
Private/commercial insurance	13.5	13.1	0.702	0.012	10.1	10.1	0.943	0.011	5.2	5.0	0.825	0.036
Medicaid	83.1	83.5	0.698		84.9	85.2	0.715		93.5	94.1	0.615	
Other insurance (ref)	3.4	3.3	0.936		5.0	4.7	0.614		1.3	0.9	0.489	
Married	39.1	38.4	0.635	0.014	39.4	39.0	0.705	0.008	14.4	12.3	0.199	0.063
Cohabiting	58.5	58.6	0.906	0.003	63.6	64.0	0.735	0.007	50.6	49.6	0.698	0.019
Primipara	51.9	52.3	0.794	0.008	40.8	39.8	0.376	0.019	34.5	33.7	0.720	0.017
Pre-existing health risk^1^	9.8	8.4	0.087	0.050	10.7	9.7	0.123	0.034	16.5	13.8	0.119	0.076

HSP: Health Start Program; SD: Standardized Difference

1. Pre-existing health risks defined as presence of pre-existing (non-gestational) diabetes and hypertension, and/or previous preterm birth

All models control for median income at zip code level, county of residence, and birth year. Subgroup matching models may include additional interactions between controls to achieve baseline equivalence

Of the 961,926 total births reported in Arizona between 2006 and 2016 approximately 0.75% were by women who participated in Health Start. Compared to statewide averages, Health Start participants were more likely to be teen parents (20% vs. 9.9%), more likely to have less than high school/GED education (32.8% vs. 21.7%), more likely to identify as Latina (59.1% vs. 41.9%) and American Indian (11.8% vs. 6.0%), more likely to have been born in Mexico (27.9% vs. 18.8%), more likely to be insured by Medicaid (82.5% vs. 53.8%), less likely to be married (37.8% vs. 54.5%) or cohabitating (62.6% vs. 75.6%), and have higher pre-existing health risks (11.3% vs. 8.2%) (Table 3a).

Tables 3a-3c also show that baseline equivalence between the Health Start cohorts and their matched controls was largely achieved. All SDs were < 0.05 for the full Health Start cohort and the primipara and less-than-high-school subgroups, meeting the strict baseline equivalence criteria. For the Latina, American Indian, teens, and rural border county subgroups additional adjustments were required. Per WWC guidelines, we used logistic regression models that included Health Start participation and all variables used in the matching models, with software-generated weights (the full results from these regressions are available on request).

### Main Findings

Tables 4a and 4b report the unadjusted propensity-score matched, and regression-adjusted propensity-score matched odds ratios associated with Health Start participation, along with the 95% confidence intervals. For consistency we describe the adjusted results here. Health Start participants were generally more likely to report both any and adequate PNC compared to the matched control group. Compared to the matched controls, participants were 24% more likely to receive any prenatal care (OR 1.24, 95%CI 1.02–1.50 ) and 8% more likely to receive adequate prenatal care (OR 1.08, 95%CI 1.01–1.16). These program effects are higher among specified subgroups, reported below.

**Table 3c:** Matching Results (Baseline Equivalence) for Health Start Participants: Less than High School & Teens Subgroups

		**Less than High School Education**								**Teens (under age 20)**							
		HSP		Matches		p-value		SD		HSP		Matches	p-value		SD		
N (Unweighted)		2,351		18,408						1,260		6,832					
**Maternal age**																	
Age < 20		30.8		30.3		0.681		0.029		100.0		100.0	1.000		0.000		
Age 20–24		27.1		26.8		0.793				0.0		0.0					
Age 25–30 (ref)		21.1		20.8		0.802				0.0		0.0					
Age > 30		20.9		22.1		0.320				0.0		0.0					
**Race/ethnicity**																	
White		14.6		14.5		0.901		0.020		22.2		22.7	0.775		0.058		
American Indian		11.1		10.7		0.607				12.5		12.2	0.809				
Latina		70.1		70.5		0.799				62.3		63.0	0.711				
Other race/ethnicity (ref)		4.1		4.4		0.665				2.9		2.1	0.161				
**Maternal nativity**																	
US (ref)		58.6		58.1		0.745		0.032		83.3		85.6	0.111		0.073		
Mexico		38.5		38.5		0.952				16.1		14.1	0.165				
Outside US		2.8		3.4		0.276				0.6		0.3	0.247				
**Maternal education**																	
Less than high school (ref)		100.0		100.0		1.000		0.000		57.5		57.8	0.878		0.044		
High school/GED		0.0		0.0						34.8		34.6	0.933				
Some post-secondary		0.0		0.0						7.3		7.0	0.757				
4-year degree or more		0.0		0.0						0.0		0.0	0.451				
Education missing		0.0		0.0						0.3		0.4	0.755				
**Insurance/payer**																	
Private/commercial insurance		3.3		3.3		1.000		0.022		6.0		5.8	0.800		0.017		
Medicaid		93.3		93.7		0.595				90.6		91.0	0.679				
Other insurance (ref)		3.4		3.1		0.460				3.4		3.2	0.738				
Married		28.8		27.4		0.270		0.032		11.7		9.5	0.070		0.072		
Cohabiting		55.8		54.8		0.519		0.026		41.8		40.6	0.544		0.030		
Primipara		35.2		35.8		0.670		0.012		80.8		83.6	0.068		0.073		
Pre-existing health risk^1^		10.1		9.6		0.557		0.017		3.8		4.5	0.370		0.036		

HSP: Health Start Program; SD: Standardized Difference

1. Pre-existing health risks defined as presence of pre-existing (non-gestational) diabetes and hypertension, and/or previous preterm birth

All models control for median income at zip code level, county of residence, and birth year. Subgroup matching models may include additional interactions between controls to achieve baseline equivalence

**Table 4a:** Unadjusted and Adjusted Odds-Ratio Effects of Health Start Participation on Any and Adequate Prenatal Care

	Any Prenatal Care (vs. no Prenatal Care)				Adequate/Adequate Plus Prenatal Care (vs. Intermediate and Inadequate Prenatal Care)			
	Unadjusted		Regression Adjusted (Logistic)		Unadjusted		Regression Adjusted (Logistic)	
**Health Start Population**	OR	95% CI^1^	OR	95% CI	OR	95% CI^1^	OR	95% CI
Statewide	1.23*	1.04, 1.46	1.24*	1.02, 1.50	1.08**	1.02, 1.15	1.08*	1.01, 1.16
Primipara	1.58**	1.16, 2.15	1.64**	1.13, 2.38	1.16**	1.06, 1.27	1.18**	1.05, 1.32
Rural border counties	1.32	1.00, 1.74	1.45*	1.05, 1.98	1.17**	1.05, 1.30	1.18**	1.05, 1.33
Latina	1.16	0.96, 1.41	1.17	0.94, 1.47	1.06	0.99, 1.14	1.07	0.98, 1.17
American Indian	2.05*	1.14, 3.70	2.22*	1.07, 4.60	1.05	0.89, 1.25	1.05	0.86, 1.29
Less than high school	1.25	0.98, 1.59	1.28	0.97, 1.69	1.12*	1.02, 1.24	1.13*	1.00, 1.27
Teen mothers (age < 20)	1.53*	1.07, 2.19	1.58*	1.02, 2.45	1.27***	1.11, 1.46	1.31**	1.11, 1.55

1. Unadjusted Confidence interval based on estimated propensity score

*** p < .001; ** p < .01; * p < .05

**Table 4b:** Unadjusted and Adjusted Odds-Ratio Effects of Health Start Participation on Any and Adequate Prenatal Care

	Adequate Plus Prenatal Care (vs. Adequate, Intermediate, or Inadequate Prenatal Care)				Inadequate Prenatal Care (vs. Intermediate, Adequate, or Adequate Plus Prenatal Care)			
	Unadjusted		Regression Adjusted (Logistic)		Unadjusted		Regression Adjusted (Logistic)	
**Health Start Population**	OR	95% CI^1^	OR	95% CI	OR	95% CI^1^	OR	95% CI
Statewide	1.05	0.98, 1.13	1.05	0.97, 1.13	0.99	1.06, 0.92	1.00	0.92, 1.08
Primipara	1.11	1.00, 1.24	1.10	0.97, 1.24	0.87*	0.97, 0.78	0.86*	0.76, 0.98
Rural border counties	1.10	0.97, 1.25	1.08	0.93, 1.24	0.85**	0.96, 0.76	0.84*	0.74, 0.96
Latina	1.12*	1.02, 1.22	1.12*	1.01, 1.25	0.99	1.08, 0.92	1.00	0.90, 1.10
American Indian	1.07	0.86, 1.34	1.04	0.80, 1.33	1.14	0.94, 1.38	1.15	0.92, 1.45
Less than high school	1.16*	1.03, 1.32	1.18*	1.02, 1.36	1.01	0.91, 1.13	1.03	0.90, 1.17
Teen mothers (age < 20)	1.03	0.87, 1.23	1.03	0.85, 1.26	0.88	1.02, 0.75	0.87	0.73, 1.05

1. Unadjusted Confidence interval based on estimated propensity score.

*** p < .001; ** p < .01; * p < .05

### Any Prenatal Care

As described, women who participated in Health Start during 2006–2016 had a higher rate of any PNC as a group compared to their matched controls. This is nominally true for all subgroups (Table 4a). American Indian participants were 122% more likely to receive any PNC (OR 2.22, 95%CI 1.07–4.60). Primipara participants (OR 1.64, 95%CI 1.13–2.38), teen participants (OR 1.58, 95%CI 1.02–2.45), and participants in rural border counties (OR 1.45, 95%CI 1.05–1.98) were also all more likely to have reported any PNC compared to their matched controls. Although rates were higher for Latina women and women with less than high school education, the differences were not statistically significant.

### Adequate Prenatal Care

We found that all subgroups of women who participated in Health Start during 2006–2016 had higher rates of (at least) Adequate PNC (vs. Intermediate or Inadequate PNC) compared to matched controls (Table 4a). Teen participants were 31% more likely to receive adequate PNC (OR 1.31, 95%CI 1.11–1.55) and both primipara participants (OR 1.18, 95%CI 1.05–1.32) and rural border county participants (OR 1.18, 95%CI 1.05–1.33) were 18% more likely, compared to their matched controls. Although adequate PNC rates were higher for Latina and American Indian participants, the differences were not statistically significant.

### Adequate Plus and Inadequate Prenatal Care

For completeness, Table 4b reports the impact of Health Start participation on the two endpoints of the APNCU Index: Adequate Plus (the highest) and Inadequate (the lowest). While all subgroups of women who participated in Health Start during 2006–2016 had nominally higher rates of Adequate Plus PNC compared to their matched controls, only among Latinas and women with less than high school education were the differences statistically significant. At the other end of the index, only rural border county and primipara participants were significantly less likely to report Inadequate PNC, compared to their matched control groups.

## Discussion

Results of our study show that participation in Health Start significantly improves initiation and utilization of prenatal care, as a whole and among a number of diverse subgroups of participants including American Indian women, women residing in rural Arizona-Mexico border counties, women with less than a high school/GED education, teens, and primipara women.

Health Start CHWs receive Core Competency and maternal and child-specific training designed to deliver access to and appropriate utilization of PNC. These trainings contribute to efforts which provide both medical and non-medical supports known to address the social determinants of PNC generally (Redding et al., 2015), especially among ethno-racially diverse women (Pan et al., 2020; Redding et al., 2015; Tough et al., 2006). Community embeddedness and outreach to women preconception and prenatally may contribute to their ability to provide timely perinatal education known to enhance likelihood of PNC utilization.

More broadly, among studies of CHW MCH home visitors, CHWs often provide practical, non-medical, peer-like support in women’s homes, focusing on social support, practical assistance, supporting optimal prenatal health, and connections to community resources (Tough et al., 2006). In the US, CHWs are known to contribute to the initiation of early and appropriate PNC among health disparate populations, consistent with our results (Coughlin et al., 2013; Rosenbach et al., 2010; Rossouw et al., 2019; Williams et al., 2017). These findings build on earlier research which showed that Health Start participation was associated with positive effects on birth outcomes (Sabo et al., 2021; Hussaini et al., 2011). To this, our study adds evidence supporting the efficacy of CHW promotion of the importance of PNC, which in turn supports the efficacy of CHW-led home visiting interventions generally and among demographically diverse populations of women as well. Moreover, most empirical studies of prenatal home visitation effectiveness examine programs which employ licensed health professionals (e.g. nurses and social workers) in combination with a peer educator or CHW (Flynn et al., 2008; Pan et al., 2020; Roman et al., 2014; Williams et al., 2017). In contrast, the Arizona Health Start Program is one of the few US-based programs where CHWs are the primary interventionists, which can now be associated with increased PNC access and utilization (Coughlin et al., 2013; Rosenbach et al., 2010).

The positive program effects among American Indian women, women in Arizona-Mexico border counties, and teens is particularly promising. The Arizona Health Start Program has prioritized partnering with county health departments and community-based health centers with strong positive ties within border regions and tribal nations. Health Start employs local Latina and American Indian CHWs as MCH home visitors to promote prenatal healthcare access and education in their communities through cultural and traditional knowledge and practices. Health Start tailors home visitation to directly meet community needs. This includes navigation of the unique Indian Health Service and tribally-operated healthcare systems, coordinating binational systems of care in the border region, and working closely with alternative schools for teen parents. CHWs provide trusted social support, preconception health education (including pregnancy prevention), and coordinate care services.

Ultimately, Health Start CHWs reflect the communities and women they serve. The CHW-led home visiting activities exceed the provision of perinatal health education; CHWs provide camaraderie, critical links to the health and social service systems foundational to utilization of PNC services, and social support to navigate the social determinants of MCH. While these functions are not measured in this present study they are worth acknowledging. Despite systematic, structural, and social barriers experienced by many pregnant women in Arizona, our study demonstrates an increase in utilization of PNC services among those who participated in the Health Start Program. Future analyses of Health Start should consider the program-level mechanisms for producing these outcomes.

### Limitations

A primary limitation of the present study is our reliance on birth certificate data for the creation of our PNC measures. These records use a combination of medical records and self-report to quantify PNC encounters and as a result this information can be inaccurate (Gregory et al., 2019). Consequently, our results are likely to be subject to measurement-error bias. A more general limitation of matching methods is the assumption that participation in the intervention is fully explained by observable characteristics. In fact, Health Start eligibility includes one or more social risk factors (e.g. domestic violence, lack of social support, inconsistent employment), which are not collected on birth certificates and thus not included in our analyses. To the extent that these programmatic factors are associated with reduced engagement with PNC or over-represented in the Health Start population, our results likely underestimate the impact of Health Start, while participant motivation and other individual level factors may mean the effects are over-stated. Additionally, while less than 1% of Health Start women reported participating in other home visiting programs, interaction with other such programs could bias estimated treatment effects attributed solely to Health Start. Finally, while matching methods developed control groups with similar baseline characteristics, the results are reflective of Arizona’s unique demography and may have limited external validity for other populations.

### Conclusions for Practice

This research contributes to emerging evidence of the impact of CHWs in coordinated systems of PNC and may provide guidance to policymakers, practitioners, and administrators efficiently target outreach and enrollment resources to potentially reduce the cost burden to health systems. In Arizona, this study provides strong evidence for sustainable investment for rural, tribal, and border CHW home visitation sites to ensure MCH care continuity and equity among ethno-racially and geographically diverse women. Future evaluation of Health Start will investigate the program impact on additional maternal and child health outcomes, with particular focus on families insured by Medicaid. Targeted investment in Health Start to engage with nulliparous young women (including adolescents) across Arizona could improve reproductive health planning, improve preconception health, and reduce unintended pregnancy through early and adequate engagement with prenatal care.

## Electronic Supplementary Material

Below is the link to the electronic supplementary material.

Supplementary Material 1

## Data Availability

At the conclusion of our study, de-identified data may be available upon request.

## Code Availability

Available upon request.

## References

[CR2] American Academy of Pediatrics and The American College of Obstetricians and Gynecologists (2017). *Guidelines for perinatal care: Eighth edition*. https://www.acog.org/clinical-information/physician-faqs/-/media/3a22e153b67446a6b31fb051e469187c.ashx

[CR3] Arizona Department of Health Services [ADHS] (2015). *Arizona maternal child health needs assessment*. https://azdhs.gov/documents/prevention/womens-childrens-health/reports-fact-sheets/title-v/needs-assessment2015.pdf

[CR4] Arizona Department of Health Services [ADHS] (2016). *Arizona health status and vital statistics 2016 annual report*. https://pub.azdhs.gov/health-stats/report/ahs/ahs2016/index.php?pg=state

[CR5] Clements KM, Barfield WD, Ayadi MF, Wilber N (2007). Preterm birth-associated cost of early intervention services: an analysis by gestational age. Pediatrics.

[CR6] Coughlin RL, Kushman EK, Copeland GE, Wilson ML (2013). Pregnancy and birth outcome improvements for american indians in the healthy start project of the inter-tribal council of Michigan, 1998–2008. Maternal and Child Health Journal.

[CR7] Dehejia R, Wahba S (2002). Propensity score-matching methods for nonexperimental causal studies. Review of Economics and Statistics.

[CR8] Flynn L, Budd M, Modelski J (2008). Enhancing resource utilization among pregnant adolescents. Public Health Nursing.

[CR9] HomVEE. (n.d.). *Review process*. US Department of Health and Human Services. https://homvee.acf.hhs.gov/review-process/Overview

[CR10] Hussaini SK, Holley P, Ritenour D (2011). Reducing low birth weight infancy: Assessing the effectiveness of the Health Start program in Arizona. Maternal and Child Health Journal.

[CR11] Johnson MB (2020). Prenatal care for American Indian women. The American Journal of Maternal/Child Nursing.

[CR12] Kotelchuck M (1994). An evaluation of the Kessner Adequacy of Prenatal Care Index and a proposed Adequacy of Prenatal Care Utilization Index. American Journal of Public Health.

[CR13] Gregory, E. C. W., Martin, J. A., Argov, E. L., & Osterman, M. J. K. (2019). Assessing the quality of medical and health data from the 2003 birth certificate revision: Results from New York City. *National Vital Statistics Reports, 68*(8), no.1120 32501201

[CR14] McDonald JA, Argotsinger B, Mojarro O, Rochat R, Amatya A (2015). First trimester initiation of prenatal care in the US-Mexico border region. Medical care.

[CR15] National Partnership for Women and Families [NPWF] (2019). *American Indian and Alaska Native Women’s Maternal Health: Addressing the crisis*. National Partnership. https://www.nationalpartnership.org/our-work/resources/health-care/maternity/american-indian-and-alaska.pdf

[CR17] Office of Minority Health. (n.d.). *Minority Population Profiles*. US Department of Health and Human Services. https://minorityhealth.hhs.gov/omh/browse.aspx?lvl=2&lvlID=26

[CR18] Pan, Z., Veazie, P., Sandler, M., Dozier, A., Molongo, M., Pulcino, T., Parisi, W., & Eisenberg, K. W. (2020). Perinatal health outcomes following a community health worker-supported home-visiting program in Rochester, New York, 2015–2018. *American Journal of Public Health, 110*(7), 1031–1033 10.2105/AJPH.2020.305655 PMC7287532 32437282

[CR19] Raglan GB, Lannon SM, Jones KM, Schulkin J (2016). Racial and ethnic disparities in preterm birth among American Indian and Alaska Native women. Maternal and Child Health Journal.

[CR20] Redding S, Conrey E, Porter K, Paulson J, Hughes K, Redding M (2015). Pathways community care coordination in low birth weight prevention. Maternal and Child Health Journal.

[CR21] Roman L, Raffo JE, Zhu Q, Meghea CI (2014). A statewide Medicaid enhanced prenatal care program: Impact on birth outcomes. JAMA Pediatrics.

[CR22] Rosenbach M, O’Neil S, Cook B, Trebino L, Walker DK (2010). Characteristics, access, utilization, satisfaction, and outcomes of Healthy Start participants in eight sites. Maternal and Child Health Journal.

[CR23] Rosenthal, E., Rush, C., & Allen, C. (2016). Understanding scope and competencies: A contemporary look at the United States community health worker field. Resource document. The Community Health Worker Core Consensus Project. https://www.c3project.org/resources. Accessed 14 July 2022

[CR24] Rossouw L, Burger RP, Burger R (2019). An incentive-based and community health worker package intervention to improve early utilization of antenatal care: Evidence from a pilot randomised controlled trial. Maternal and Child Health Journal.

[CR25] Sabo S, Allen CG, Sutkowi K, Wennerstrom A (2017). Community Health Workers in the United States: Challenges in identifying, surveying, and supporting the workforce. American Journal of Public Health.

[CR26] Sabo, S., Butler, M., McCue, K., Wightman, P., Pilling, V., Celaya, M., & Rumann, S. (2019). Evaluation protocol to assess maternal and child health outcomes using administrative data: A community health worker home visiting programme.BMJ Open, 9(12), e031780 10.1136/bmjopen-2019-031780 PMC6924704 31826891

[CR27] Sabo, S., Wightman, P., McCue, K., Butler, M., Pilling, V., Jimenez, D., Celaya, M., & Rumann, S. (2021). Accepted for publication on 12 Apr. 2021). Addressing maternal and child health equity through a community health worker home visiting intervention to reduce low birthweight: Retrospective quasi-experimental study of the Arizona Health Start Program. *BMJ Open*, *11*(6), e045014 10.1136/bmjopen-2020-045014 PMC8211081 34135037

[CR28] Selchau K, Babuca M, Bower K, Castro Y, Coakley E, Flores A, Garcia J, Reyes MLF, Rojas Y, Rubin J, Samuels D, Shattuck L (2017). First trimester prenatal care initiation among Hispanic women along the U.S.-Mexico border. Maternal and Child Health Journal.

[CR29] Shaffer CF (2002). Factors influencing the access to prenatal care by Hispanic pregnant women. Journal of the American Academy of Nurse Practitioners.

[CR30] Tough SC, Johnston DW, Siever JE, Jorgenson G, Slocombe L, Lane C, Clarke M (2006). Does supplementary prenatal nursing and home visitation support improve resource use in a universal health care system? A randomized controlled trial in Canada. Birth.

[CR31] Vintzileos A, Ananth C, Smulian J, Scorza W, Knuppel R (2002). The impact of prenatal care in the United States on preterm births in the presence and absence of antenatal high-risk conditions. American Journal of Obstetrics and Gynecology.

[CR32] What Works Clearinghouse (2020). *What Works Clearinghouse Standards Handbook, Version 4.1*.

[CR33] Williams CM, Cprek S, Asaolu I, English B, Jewell T, Smith K, Robl J (2017). Kentucky Health Access Nurturing Development Services home visiting program improves maternal and child health. Maternal and Child Health Journal.

