# Supplementary material for: Impact of a Community Health Worker (CHW) Home Visiting Intervention on Any and Adequate Prenatal Care Among Ethno-Racially Diverse Pregnant Women of the US Southwest
Source: Matern Child Health J. 2022 Oct 21;26(12):2485–95. doi: 10.1007/s10995-022-03506-2 (PMC9747829; doi:10.1007/s10995-022-03506-2)
Supplement: Supplementary file 1 — Supplementary Material 1 [file 10995_2022_3506_MOESM1_ESM.docx]

Supplementary Tables

| **Table 5a: Average Treatment-on-the-Treated (ATT) Effects of Health Start Participation on Any and Adequate Prenatal Care** | | | | | | | | | | |
| --- | --- | --- | --- | --- | --- | --- | --- | --- | --- | --- |
|  | **Any Prenatal Care**  (vs no Prenatal Care) | | | | | **Adequate/Adequate Plus Prenatal Care**  (vs Intermediate and Inadequate Prenatal Care) | | | | |
| **Health Start Population** | HSP % | Matched % | ATT | 95% CI* | p-value* | HSP % | Matched % | ATT | 95% CI* | p-value* |
| Statewide | **97.1** | **96.5** | **0.64** | **0.11, 1.16** | **0.017** | **63.3** | **61.4** | **1.85** | **0.46, 3.24** | **0.009** |
| Primipara | **98.3** | **97.3** | **0.96** | **0.30, 1.61** | **0.004** | **66.7** | **63.3** | **3.38** | **1.24, 5.51** | **0.002** |
| Rural border counties | 96.6 | 95.6 | 1.04 | -0.00, 2.07 | 0.051 | **61.5** | **57.8** | **3.72** | **1.21, 6.22** | **0.004** |
| Latina | 96.1 | 95.5 | 0.61 | -0.17, 1.39 | 0.126 | 60.6 | 59.1 | 1.48 | -0.29, 3.25 | 0.101 |
| American Indian | **98.5** | **96.9** | **1.57** | **0.28, 2.85** | **0.017** | 56.9 | 55.6 | 1.29 | -2.96, 5.55 | 0.551 |
| Less than high school | 95.4 | 94.4 | 1.06 | -0.09, 2.22 | 0.071 | **55.9** | **53.1** | **2.85** | **0.38, 5.32** | **0.024** |
| Teen mothers (age<20) | **96.7** | **95.1** | **1.65** | **0.26, 3.03** | **0.020** | **59.7** | **53.8** | **5.86** | **2.45, 9.27** | **0.001** |
| HSP: Health Start Program  * ATT Confidence interval and p-value based on estimated propensity score. | | | | | | | | | | |

| **Table 5b: Average Treatment-on-the-Treated (ATT) Effects of Health Start Participation on Inadequate and Adequate Plus Prenatal Care** | | | | | | | | | | |
| --- | --- | --- | --- | --- | --- | --- | --- | --- | --- | --- |
|  | **Adequate Plus Prenatal Care**  (vs Adequate, Intermediate, or Inadequate Prenatal Care) | | | | | **Inadequate Prenatal Care**  (vs Intermediate, Adequate, or Adequate Plus Prenatal Care) | | | | |
| **Health Start Population** | HSP % | Matched % | ATT | 95% CI* | p-value* | HSP % | Matched % | ATT | 95% CI* | p-value* |
| Statewide | 23.1 | 22.2 | 0.93 | -0.29, 2.15 | 0.135 | 23.8 | 24.0 | -0.21 | -1.44, 1.01 | 0.734 |
| Primipara | 24.5 | 22.6 | 1.87 | -0.07, 3.80 | 0.059 | **21.3** | **23.7** | **-2.43** | **-4.31, -0.55** | **0.011** |
| Rural border counties | 20.9 | 19.4 | 1.52 | -0.57, 3.62 | 0.154 | **26.5** | **29.8** | **-3.22** | **-5.54, -0.89** | **0.007** |
| Latina | **22.2** | **20.3** | **1.87** | **0.37, 3.37** | **0.015** | 26.9 | 27.0 | -0.12 | -1.75, 1.51 | 0.886 |
| American Indian | 18.9 | 17.9 | 1.02 | -2.31, 4.36 | 0.548 | 27.0 | 24.5 | 2.46 | -1.22, 6.14 | 0.191 |
| Less than high school | **22.1** | **19.6** | **2.51** | **0.49, 4.54** | **0.015** | 31.3 | 31.0 | 0.29 | -2.02, 2.61 | 0.803 |
| Teen mothers (age<20) | 20.9 | 20.4 | 0.51 | -2.31, 3.33 | 0.722 | 27.8 | 30.4 | -2.64 | -5.80, 0.51 | 0.100 |
| HSP: Health Start Program  * ATT Confidence interval and p-value based on estimated propensity score. | | | | | | | | | | |
